# Acupuncture for Hashimoto thyroiditis: study protocol for a randomized controlled trial

**DOI:** 10.1186/s13063-021-05036-8

**Published:** 2021-01-21

**Authors:** Shanze Wang, Jiping Zhao, Weimei Zeng, Wanqing Du, Tenghui Zhong, Hui Gao, Yi Xiao, Chao Yang

**Affiliations:** 1 The Department of Acupuncture, Dongzhimen Hospital, Beijing University of Chinese Medicine, Beijing, 100700 China; 2 Department of Encephalology, Xiyuan Hospital, China Academy of Chinese Medical Sciences, Beijing, China

**Keywords:** Hashimoto thyroiditis, Acupuncture, Randomized controlled trials, Penetration needling

## Abstract

**Background:**

The incidence rate of Hashimoto thyroiditis (HT) has gradually increased in recent years. There has been no specific etiological treatment for HT. Even though with normal level of thyroid hormone, the patients may still suffer from various clinical symptoms, such as anterior neck discomfort, fatigue, and mood swings, which seriously impair their quality of life. Acupuncture has long been used in the treatment of thyroid diseases, but there has been no related standardized clinical study as of today. This study aims to assess the feasibility, efficacy, and safety of acupuncture for HT.

**Methods:**

This is a randomized, black-controlled assessor-blinded pilot trial. A total of 60 patients will be recruited and divided into the experimental group (*n* = 30) or the control group (*n* = 30). The experimental group will undergo acupuncture therapy (penetration needling of Hand-Yangming meridian, PNHM) for 16 weeks, followed by a 16-week follow-up period, and the control group will first go through an observation period for 16 weeks, followed by a 16-week compensation PNHM therapy. The primary outcome will be the change of the concentrations of anti-thyroperoxidase antibodies (TPOAb), antithyroglobulin antibodies (TgAb), and thyroid hormone, including total thyroxine (FT_4_), free thyroxine (FT_3_), and thyroid-stimulating hormone (TSH). The secondary outcome measurements include the thyroid-related quality of life questionnaire short-form (ThyPRO-39), The Mos 36-item Short Form Health Survey (SF-36), and Hospital Anxiety and Depression Scale (HAD). Data collection will be performed before the start of the study (the baseline assessment) and at weeks 8, 16, 24, and 32.

**Discussion:**

The study is designed to assess the feasibility and effectiveness of PNHM in reducing the thyroid antibody level and improving the quality of life of HT patients with hypothyroidism or subclinical hypothyroidism. Results of this trial will assist further analyses on whether the acupuncture treatment can alleviate symptoms for patients with HT.

**Trial registration:**

Acupuncture-Moxibustion Clinical Trial Registry AMCTR-IOR-19000308 (ChiCTR1900026830). Registered on 23 October 2019.

## Background

Hashimoto’s thyroiditis (HT), also known as chronic lymphocytic thyroiditis, has become one of the most common endocrine disorders and the most common leading cause of hypothyroidism in recent years, as a result of its gradually increasing incidence rate. The cause of HT is unclear and the diagnoses are mainly based on the positive serum anti-thyroperoxidase antibodies (TPOAb). According to epidemiological studies from different countries, the positive rate of the TPOAb in the population reportedly varies from 8.4 to 17% worldwide [1–4], increasing with age. Among people with elevated TPO antibodies, about 2.5% progress to clinical hypothyroidism each year [5].

HT is a chronic autoimmune disease; after a transient hyperthyroidism that may occur in the early stage, it gradually develops to hypothyroidism in the final stage caused by the autoimmune destruction of the thyroid. Abnormal thyroid function manifests as metabolic abnormalities, thyroid symptoms, and various systemic symptoms. Thyroid symptoms include goiter and anterior neck discomfort. One study stated that the symptom of goiter is not severe in HT patients, but the neck compression symptoms caused by goiter is significant [6]. Systemic symptoms include celiac disease, fatigue, and mood swings [7–9]. The risk of miscarriage and infertility increased in the young women with HT, and thyroid hormone supplementation is not effective in relieving this situation [10]. It was found that HT patients with normal thyroid function may suffer from various clinical symptoms which reduce the quality of life and this may be positively correlated with high thyroid antibody concentrations [11, 12].

It is widely believed that proper intake of nutrients such as iodine, selenium, and iron is beneficial to HT patients [13]. However, there is still no specific etiological treatment for HT. Most studies have mentioned that selenium supplementation can reduce thyroid antibody levels. Although the antibody level is significantly reduced in selenium supplementation, which lasted for more than 3 months, may rebound after the end of treatment [14, 15]. However, some studies have different results that selenium supplementation provides no effect in reducing thyroid antibody level [16–18]. In addition, a gluten-free protein diet may be beneficial for HT patients, but one study stated briefly that this diet plan resulted in a huge impact on the life quality of HT patients more than the disease itself. For the consensus that supplementation of levothyroxine (L-T4) is viable for the treatment of HT patients with subclinical hypothyroidism or clinical hypothyroidism, the starting point and end point of oral thyroid hormone treatment still need confirmation. Some studies indicate that oral thyroid hormone can relieve clinical symptoms of patients when TSH exceeds 10 mIU/l [19]. Different approaches for the treatment of HT require further exploration.

Based on the records from ancient acupuncture books, we find that acupoints on the Yangming meridian have been extensively used in treating various thyroid diseases [20]. Acupoints on the front neck and upper arm, especially at the front edge of the deltoid muscle of the upper arm, were commonly chosen by ancient traditional Chinese medicine (TCM) physicians in the treatment of goiter. This reflects the possible physiological and pathological connection between the thyroid and the upper arms, which further indicates the clinical value of acupuncture on the upper arms in the treatment of thyroid diseases. In modern times, Professor Le-ting Wang, a famous TCM acupuncture physician, is experienced in the treatment of gland diseases of the cervix, including mumps, lymph node tuberculosis, and thyroid disease by applying penetration needling at specific acupoints such as Qu Chi (LI11) and Bi Nao (LI14). Previous studies suggest that acupuncture, as a therapy of complementary and alternative medicine, can bring benefits to patients with thyroid disease [21]. However, there are few randomized controlled trials and few research designs for HT currently. Some important design details in these published studies, such as randomized method and acupuncture scheme, are also insufficient. Therefore, we design the protocol for an RCT to initially explore the feasibility and clinical effectiveness of the treatment of HT with penetration needling of Hand-Yangming meridian (PNHM).

## Methods

### Objective

The objective is to assess the feasibility and effectiveness of PNHM in reducing the thyroid antibody level and improving the life quality of HT patients with hypothyroidism or subclinical hypothyroidism.

### Study design

**Fig. 1:**
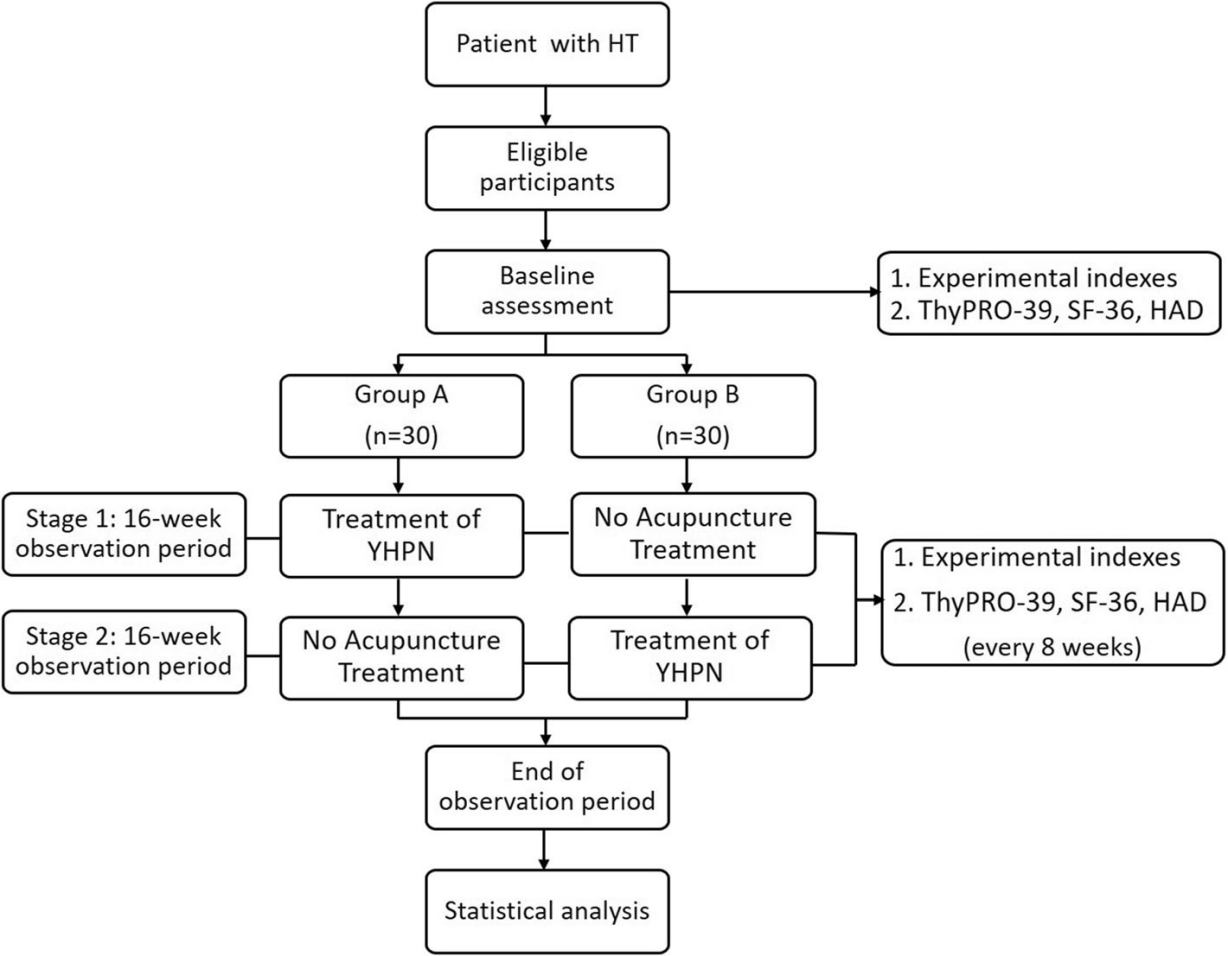
Trial flow diagram

**Fig. 2:**
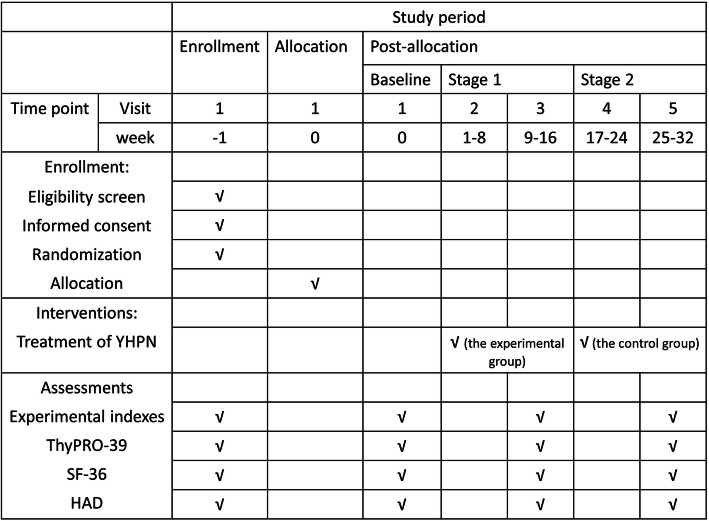
The time point of assessment

This study is a randomized, blank-controlled, parallel-group clinical trial. Participants will be assigned to the experimental group or the control group. Study period for both groups lasts for 33 weeks. Eligible participants will be randomly divided into the experimental group and the control group with a ratio of 1:1. The experimental group will undergo acupuncture therapy (PNHM) for 16 weeks, followed by a 16-week follow-up period and the control group will first enter an observation period for 16 weeks, followed by a 16-week compensation acupuncture therapy (PNHM). Assessments will be conducted at baseline and 8, 16, 24, and 32 weeks after randomization. The flowchart is shown in Fig. 1 and the timepoint of assessment is shown in Fig. 2.

### Participants

#### Diagnosis criteria

The diagnosis criteria is a combination of the presence of serum antibodies against thyroid antigens and appearance on thyroid sonogram according to a review and the diagnosis criteria in Chinese clinical guidelines [22, 23]. If thyroid ultrasound indicates diffuse enlargement of the thyroid and reduced echogenicity of glands in a patient with elevated serum thyroid-associated antibody, the clinical diagnosis of HT can be established.

#### Inclusion criteria

Eligible participants should meet the following requirements:

1. Confirmed clinical diagnosis of Hashimoto’s thyroiditis;
2. Age between 18 and 70 years old;
3. Serum thyroid hormone was within the normal level, and regular administration of exogenous levothyroxine for the past 1 month; and
4. The informed consent signed.

#### Exclusion criteria

Individuals will be excluded from the study if they meet any of the following criteria:

1. Thyroid-stimulating hormone receptor antibody (TRAb) positive, and be suspected of combining Graves’ disease or Hashimoto’s hyperthyroidism;
2. Highly suspected thyroid malignancy;
3. Highly suspected subacute thyroiditis;
4. A history of thyroid surgery;
5. Took medicine that may influence antibody concentration (such as selenium supplementation, some Chinese herbal medicine) except for exogenous thyroid hormone in the past 1 month;
6. Severe systemic disease including cardiovascular, cerebrovascular, liver, kidney, and blood system diseases;
7. A history of mental illness;
8. Pregnancy or lactation women;
9. Needle-phobic patients; and
10. Existing local infection or severe skin lesions near the needle site.

#### Withdrawal criteria

Participants will be withdrawn from the study if the following occurs:

1. Less than 75% of total treatment (i.e., participating in fewer than 36 of the 48 scheduled treatment sessions)
2. Participant’s decision to withdraw from the study at any time for any reason; and
3. Occurrence of any unexpected serious side effects.

### Study setting and recruitment

This study will be performed at Dongzhimen Hospital Affiliated to Beijing University of Chinese Medicine between October 2019 and June 2021. A total of 60 patients will be recruited through advertisements placed on social media and community centers. A 1-week baseline assessment will be needed before randomization. During the baseline assessment period, volunteers will provide relevant medical records of thyroid diseases and complete clinical evaluation. Subjects who meet the acceptance criteria and agree to join the study will further complete the baseline data and wait for the result of random grouping allocation. Subjects who cannot join the group for any reason will be given a full explanation and health guidance.

### Randomization and allocation concealment

The random number sequence will be generated with SPSS software version 23 (IBM Corp., Armonk, NY, USA) by an independent researcher who will be in charge of assigning eligible participants into two groups equally and randomly. The independent researcher will not participate in patient treatment, outcome evaluation, data collection, or data analysis. Opaque envelopes containing randomized serial numbers for grouping inside with numbers in order on the cover will be prepared and kept by the independent researcher who took no part in this trial. When the eligible participants sign the informed consent form, the clinical researcher will receive an envelope according to the sequence numbers from the independent researcher and attain the information of the group allocation.

### Blinding

Because of the particularity of acupuncture, it is difficult to blind clinical researchers and participants. In this study, only two data researchers in charge of the collection, preservation, and analysis of data are blinded. Participants will provide information to the date researchers through online questionnaires without communication between both sides. So that data researchers cannot be aware of the grouping allocation. In this study, the data of each participant will be collected every 8 weeks, hence the researchers also cannot infer the grouping allocation through data information. Data researchers will be asked to speculate the grouping allocation to evaluate the implementation of the blind method.

### Sample size estimation

This study aims to evaluate the clinical effectiveness and safety of PNHM for HT. The estimation of sample size should be based on the feasibility, precision about the mean, and variance [24]. It is stipulated that the sample size of exploratory trials is 20–30 per group in China [25]. With consideration of the drop rate of 20% and the feasibility of the study, we finally plan to recruit 60 participants in total to assure the validity of the mean and effect size.

### Intervention

The intervention protocol of this study is based on the consensus of experienced acupuncture practitioners and thyroid disease specialists. Acupuncturists who hold a TCM license will perform the treatment. Participants of both groups will undergo acupuncture treatment for 16 weeks at different stages of the study. The details of the PNHM are as follows and shown in Table 1.

**Table 1:** Locations and manipulations of the acupuncture points selected in this study

Acupoints	Location	Size of needle	Manipulation
**San Jian (LI3)**	On the dorsum of the hand, in the depression radial and proximal to the second metacarpophalangeal joint.	0.25 × 40 mm	Insert the needle to a depth of 1–2 mm, adjust the direction of needle’s tip towards He Gu (LI4) and penetrate the needle horizontally within the subcutaneous tissue to a depth 0.8–1.0 cun (20.0 mm–25.0 mm). Manipulate needle to get “de-qi” sensation.
**He Gu (LI4)**	On the dorsum of the hand, radial to the midpoint of the second metacarpal bone.	0.18 × 40 mm	Same as “San Jian (LI3)”
**Qu Chi (LI11)**	On the lateral aspect of the elbow, at the midpoint of the line connecting LU5 with the lateral epicondyle of the humerus.	0.30 × 75 mm	Insert needle to a depth of 1–2 mm, adjust the direction of needle’s tip towards Bi Nao (LI14) and penetrate the needle horizontally within the subcutaneous tissue to a depth 2.5–2.8 cun (62.5 mm–70.0 mm). Manipulate needle to get “de-qi” sensation.
**Bi Nao (LI14)**	On the lateral aspect of the arm, just anterior to the border of the deltoid muscle, 7 B-cun superior to LI11	0.30 × 75 mm	Insert needle to a depth of 1–2 mm, adjust the direction of needle’s tip towards Jian Yu (LI15) and penetrate the needle horizontally within the subcutaneous tissue to a depth 2.5–2.8 cun (62.5 mm–70.0 mm). Manipulate needle to get “de-qi” sensation.
**Jian Yu (LI15)**	On the shoulder girdle, in the depression between the anterior end of lateral border of the acromion and the greater tubercle of the humerus.	0.25 × 40 mm	Insert needle obliquely to a depth of 0.8–1.5 cun (20.0 mm–37.5 mm). Manipulate needle to get “de-qi” sensation.
**Ren Ying (ST9)**	In the anterior region of the neck, at the same level as the superior border of the thyroid cartilage, anterior to the sternocleidomastoid muscle, over the common carotid artery.	0.25 × 40 mm	Insert needle perpendicularly to a depth of 0.8–1.5 cun. Manipulate needle to get “de-qi” sensation.
**Zu San Li (ST36)**	On the anterior aspect of the leg, on the line connecting ST35 with ST41, 3 B-cun inferior to ST35	0.25 × 40 mm	Insert needle perpendicularly to a depth of 1.0–1.5 cun (25.0 mm–37.5 mm). Manipulate needle to get “de-qi” sensation.

Acupoints: San Jian (LI3), He Gu (LI4), Qu Chi (LI11), Bi Nao (LI14), Jian Yu (LI15), Ren Ying (ST9), Zu San Li (ST36). All acupoints are positioned according to the WHO Standard Acupuncture Point Locations in the Western Pacific Region [26].

Needles: “Andy” disposable sterile stainless needles, size of 0.25 × 40 mm and size of 0.30 × 75 mm needles will be used in this trial.

Manipulation: With supine positions, San Jian (LI3) will be penetrated towards He Gu (LI4), Qu Chi (LI11) will be penetrated towards Bi Nao (LI14), Bi Nao (LI14) will be penetrated towards Jian Yu (LI15). Jian Yu (LI15), Ren Ying (ST9), Zu San Li (ST36) will be punctured to standardized needling depths. De-qi sensation (an irradiation feeling deemed to indicate effective needling) should be achieved in each acupoint. Every participant should take three times of treatment per week for 16 weeks, thus a total of 48 treatments, each treatment lasting for 20 min.

Penetration needling is a technique of point-through-point needling. The procedures are as follows: the needle is first being punctured into the skin to a depth of 1–2 mm, then the direction of the needle’s tip is adjusted towards the adjacent acupoint, and finally, the needle is penetrated horizontally within the subcutaneous tissue towards an appropriate depth according to the local anatomical structures.

### Outcomes and measurements

#### Primary outcomes

The primary outcome will be the change of the concentrations of serum anti-thyroperoxidase antibodies (TPOAb), serum anti-thyroglobulin antibodies (TgAb), and the concentrations of thyroid hormone, including total thyroxine (FT_4_), free thyroxine (FT_3_), and thyroid-stimulating hormone (TSH). Data will be collected before the start of the study (the baseline assessment) and at weeks 8, 16, 24, and 32.

#### Secondary outcomes

The secondary outcome measurement includes 3 scales. Data will be collected before the start of the study (the baseline assessment) and every 8 weeks in the whole process.

##### Thyroid-related quality of life questionnaire short-form (ThyPRO-39) [27, 28]

The ThyPRO is a questionnaire of 85 items, widely used to evaluate thyroid-related symptoms and health quality of life. ThyPRO-39, a shorter version of ThyPRO, is a self-assessment scale of 39 items, using Likert 5-point scale. It has 12 subscales, further divided into 3 categories. According to the extent of thyroid disease’s impact in the past 4 weeks, every question can be rated on 0, 1, 2, 3, or 4 points, respectively, not at all, a little, some, quite a bit, or very much. Scores for each subscale need to be converted into standard points according to the score table and calculation formula, and standard total score can be calculated, ranging from 0 to 100 points. A higher score indicates a worse quality of life. ThyPRO-39 is used to evaluate the influence of HT on patient quality of life. Data will be collected before the start of treatment (the baseline assessment) and at 8 and 16 weeks after randomization.

##### The Mos 36-item Short Form Health Survey (SF-36) [29, 30]

SF-36 is the most widely used quality of life assessment tool, containing 36 items, covering nine variables. For each variable item, scores are coded, summed, and transformed on to a scale from 0 (worst possible health state measured by the questionnaire) to 100 (best possible health state). Some studies use the SF-36 to reflect the quality of life of HT patients. It is found that when the thyroid function is normal, the quality of life of HT patients is negatively correlated with antibody levels [11, 12]. Therefore, in this study, SF-36 is also used as an observation indicator to evaluate the influence of acupuncture on HT patients. Data will be collected before the start of treatment (the baseline assessment) and at 8 and 16 weeks after randomization.

##### Hospital Anxiety and Depression Scale (HAD) [31]

HAD is a 14-item self-assessment scale that have been rated on a 4-poiont scale, used to identify and quantify the two most common forms of psychological disturbances in medical patients. And HT patients often have symptoms of mood swings. Data will be collected before the start of treatment (the baseline assessment) and at 8 and 16 weeks after randomization.

### Drug combination

The current therapeutic target of HT is to maintain the stability of thyroid function due to the lack of effective treatment for the cause of HT. Therefore, in this study, all participants have been taking exogenous levothyroxine (trade name: Youjiale) to treat hypothyroidism. Considering the influence on serum thyroid-related antibodies, taking medicine that may have an impact on antibodies, such as selenium preparations, will not be allowed. All drugs used by the participants will be recorded in detail, including the drug name, dosage and treatment course, and other information. Clinical researchers will judge the effect of the drug on the study results according to the specific situation.

### Adverse events

According to our earlier clinical observations, adverse events of acupuncture mainly involve bleeding, hematoma, fainting, and serious pain, with a close relevance to the subjective experience of patients and the penetration technique of acupuncturists. Operators shall be trained repeatedly to ensure the standard, effective, and safe operation. We will record any acupuncture-related adverse events that occur during the treatment and physicians will take necessary corresponding treatment measures. In case of serious adverse events, the clinical trial shall be interrupted immediately and effective treatment measures shall be taken. The whole process will be recorded in detail.

### Data collecting and monitoring

The scale of the study will be made into a form of online questionnaire, and participants will be guided how to fill in the questionnaire before after signing the informed consent. Data researchers can receive the results of the scale and blood test through the internet without communication with participants, and record the data on the case report forms (CRFs) to ensure the integrity of the RCT and protect the privacy of the participants. Data will be safekept by the data researchers of our team and monitored by the Ethics Committee of Dongzhimen Hospital, Beijing University of Traditional Chinese Medicine every 3 months.

### Statistical analysis

Data of this clinical trial will be analyzed using SPSS version 23.0 software (released 2015, IBM Corp., Armonk, NY, USA). All data will be presented as mean ± standard deviation or value and its percentage. Demographic (age, sex, course, family history, the dosage of drugs) will be compared between two groups as the baseline assessment. The data of the observation indicators are all measurement data. According to the normal test, select the appropriate parameter test or non-parameter test. A paired *t* test or Wilcoxon signed-rank test can be used for comparison before and after treatment within group. Independent sample *t* test or Wilcoxon rank-sum test can be used for comparison between groups. In two groups, all the statistical tests will be performed with bilateral tests, and *P*< 0.05 indicates the difference deemed statistically significant.

### Quality control

This clinical trial has been reviewed by experts of general surgery, acupuncture, methodology, etc. Before the start of this trial, all acupuncturists were required to participate in training of standard operation procedure (SOP) to ensure every acupuncturist is familiar with the trial scheme and process. During the time this trial is carried out, the research team will conduct workshops every 2 months to summarize the experience and evaluate the progress of the trial, clinical feasibility, and side effects. The supervisor shall regularly check the progress of the trial.

## Discussion

The current treatment of HT focuses mainly on the symptom of hypothyroidism that occurs in the later stage of the disease via orally-taken LT4 in a long-term. However, there are not yet any effective treatments for symptoms of neck pressure, fatigue, mood swings, infertility, and others. Comprehensive management methods adopted to delay the progression of the disease or reduce the degree of hypothyroidism deserve further exploration.

Acupuncture has advantages in alleviating clinical symptoms, such as neck pressure, fatigue, and mood swings, and hardly has side effects. Some studies also suggest that acupuncture can stimulate the body to produce a series of neurohumoral immune responses and as a result improve immune disorder state [32]. This suggests that acupuncture has potential therapeutic value for HT. The abovementioned acupuncture treatments can be referred to ancient acupoints for treating thyroid diseases and the experience of modern acupuncture experts. In our earlier clinical observations, this acupuncture method can improve clinical symptoms of HT patients, and some patients’ thyroid-related antibody concentrations have decreased.

Some studies suggest a positive correlation between antibody concentration and the extent of thyroid inflammatory cell infiltration [33, 34]. Thyroid antibody concentrations are also positively correlated with the quality of life of HT patients. Thus, we have chosen antibody concentration as one of the main efficacy indicators to objectively reflect the subject’s immune status. Meanwhile, we will also observe whether the TSH is further stabilized or decreased to the normal range, so as to potentially reduce the dose of FT4 with the help of acupuncture. The use of multiple symptom score scales as secondary efficacy indicators is to fully evaluate the effect of acupuncture treatment on clinical symptoms of HT patients.

The mechanism of acupuncture treatment is complicated and is completely different from the specific target of “one key to one lock” in western pharmacology. On the one hand, there is no ideal placebo-controlled method for clinical research of acupuncture [35]. On the other hand, the non-specific clinical effects caused by placebo cannot be regarded as an insignificant or unimportant part [36]. Research methods that try to strip the biological and psychological effects of acupuncture may contradict the development model of modern medical bio-psycho-social medicine. Therefore, the non-specific effects of acupuncture also require attention. In order to reflect the comprehensive effect of acupuncture treatment, we have chosen a blank control to try to observe the effect of acupuncture treatment in the presence of a placebo effect.

There are two limitations in this study. First, the sample size is small, and the main research purpose of this study is to evaluate the feasibility and clinical value of the treatment of HT by PNHM, so the research results may not accurately reflect the true clinical effect of acupuncture on HT. Second, considering the acceptance of the subject, a blank control is assigned as the control group in this study, which could not exclude the non-specific therapeutic effect or placebo effect of acupuncture. At the same time, we cannot prove the specific therapeutic effect of the selected acupoints and acupuncture methods.

### Trial status

The protocol version is 20190410, V2.0. This study is currently in the recruitment phase. The first patient was enrolled in October 2019, and recruitment is expected to be completed before November 2020. Therefore, the last participant would finish the study by June 2021.

## Data Availability

The datasets analyzed during the current study are available from the corresponding author on reasonable request.

## Abbreviations

HT: Hashimoto’s thyroiditis
TPOAb: Antithyroperoxidase antibodies
TCM: Traditional Chinese medicine
PNHM: Penetration needling of Hand-Yangming meridian
SPIRIT: Standard Protocol Items: Recommendations for Interventional Trials
STRICTA: Standards for Reporting Interventions in Clinical Trials of Acupuncture
TRAb: Thyroid-stimulating hormone receptor antibody
TgAb: Antithyroglobulin antibodies
SOP: Standard operation procedure
CRFs: Case report forms
SF-36: The Mos 36-item Short Form Health Survey
ThyPRO-39: Thyroid-related quality of life questionnaire short-form
HAD: Hospital Anxiety and Depression Scale
FT_4_: Total thyroxine
FT_3_: Free thyroxine
TSH: Thyroid-stimulating hormone
L-T4: Levothyroxine
